# Comprehensive analysis of the codon usage patterns in the envelope glycoprotein E2 gene of the classical swine fever virus

**DOI:** 10.1371/journal.pone.0183646

**Published:** 2017-09-07

**Authors:** Ye Chen, Xinxin Li, Xiaojuan Chi, Song Wang, Yanmei Ma, Jilong Chen

**Affiliations:** 1 Key Laboratory of Fujian-Taiwan Animal Pathogen Biology, College of Animal Sciences, Fujian Agriculture and Forestry University, Fuzhou, China; 2 Institute of Microbiology, Chinese Academy of Sciences (CAS), Beijing, People’s Republic of China; South China Agricultural University, CHINA

## Abstract

The classical swine fever virus (CSFV), circulating worldwide, is a highly contagious virus. Since the emergence of CSFV, it has caused great economic loss in swine industry. The envelope glycoprotein E2 gene of the CSFV is an immunoprotective antigen that induces the immune system to produce neutralizing antibodies. Therefore, it is essential to study the codon usage of the E2 gene of the CSFV. In this study, 140 coding sequences of the E2 gene were analyzed. The value of effective number of codons (ENC) showed low codon usage bias in the E2 gene. Our study showed that codon usage could be described mainly by mutation pressure ENC plot analysis combined with principal component analysis (PCA) and translational selection-correlation analysis between the general average hydropathicity (Gravy) and aromaticity **(**Aroma), and nucleotides at the third position of codons (A3s, T3s, G3s, C3s and GC3s). Furthermore, the neutrality analysis, which explained the relationship between GC12s and GC3s, revealed that natural selection had a key role compared with mutational bias during the evolution of the E2 gene. These results lay a foundation for further research on the molecular evolution of CSFV.

## Introduction

Classical swine fever (CSF) is a World Organization for Animal Health (OIE)-listed, highly contagious viral disease characterized by fever and hemorrhage. Classical swine fever virus (CSFV) can infect domestic pigs and wild boars. CSFV infections were once distributed worldwide. However, it was successfully eliminated in some countries, including the major countries in Oceania and North America and some European Union countries [1]. CSFV belongs to the *Flaviviridae* family, and is genetically and antigenically associated with other pestiviruses, such as bovine viral diarrhea virus (BVDV) and border disease virus (BDV). CSFV is an enveloped virus, and its genome is a 12.3kb to 12.5kb long single-stranded RNA, flanked with highly conserved 5'NTR and 3'NTR [2]. It has a single open reading frame that encodes 3900–4000 amino acids and can be processed to produce different viral proteins [3]. Under the action of cellular and viral proteases, this single polyprotein, which contains 3989 amino acids, can produce four structural proteins (C, Erns, E1 and E2) and eight non-structural proteins (Npro, p7, NS2, NS3, NS4A, NS4B, NS5A and NS5B) [4]. The envelope glycoprotein E2, located on the outer surface of the virus, is highly immunogenic and induces a protective neutralizing antibody in a natural host [5]. Beyond that, it has been demonstrated that the E2 protein is a virulence determinant and participates in the adsorption of the virus to the host cell [6, 7].

In addition to methionine and tryptophan, amino acids can be encoded by more than one codon because the genetic code is redundant, which is also known as synonymous codon usage. However, the use of various codons to encode amino acids is not random, and some are more frequently used, which is called codon usage bias [8]. Some RNA viruses display codon usage bias; however, the degree of deviation varies depending on the identity of the virus. For example, rubella and rotavirus show a strong codon usage bias, while SARS, FMDV and swine epidemic diarrhea virus (PEDV) show weak codon bias [9–11]. Mutation pressure, natural selection, gene length, tRNA abundance and RNA structure all affect codon usage bias [12–14]. Viruses and hosts can affect the use of codons, which may affect the survival of the virus from evolution, host defense, suitability and immune escape. In fact, synonymous triads are not randomly used, and factors such as natural selection and mutation bias are known to cause this deviation to be equivalent to the use of codons [15]. The study of codon usage patterns in viruses can provide a thorough understanding of their molecular evolution, viral gene expression regulation, and vaccine design that may require high levels of viral antigen expression to produce immunity [16].

Considering the highly contagious nature of CSFV and importance of E2 gene, it is necessary to understand the codon usage patterns of E2 gene during its evolution. In this study, we perform a genome comprehensive analysis of codon usage and the various factors that have contributed to the molecular evolution of CSFV.

## Materials and methods

### Sequence data

In this study, 140 coding sequences of the E2 gene of CSFV were retrieved from the National Center for Biotechnology (NCBI) GenBank database (https://www.ncbi.nlm.nih.gov/nucleotide/). The detailed information of the 140 sequences, including the accession number, geographical distribution of the isolated strains and time they were isolated, are listed in S1 Table.

### Recombination analysis

Before analyzing the codon usage index, the sequences were exerted to exclude the potential recombination sequences by the Recombination Detection Program 4.0 (RDP4) [17]. In the analysis, the P value cut off was 0.05 for analyses of all the selected sequences. Additionally, Bonferroni’s correction operator was selected in this study. In identifying the potential recombination of the 140 sequences, seven methods (RDP, GENECONV, MaxChi, BootScan, SiScan, 3SEQ and Chimaera) of the RDP4 were exerted in this study. The recombination was identified only when at least three of the above seven algorithms consistently conformed [18].

### Nucleotide content and codon usage composition

The nucleotide contents (A%, T%, G%, and C%) of each CSFV sequence were calculated with the software Bio Edit (version 7.0.9.0). Each nucleotide at the third position of the synonymous codon (A3%, T3%, G3%, and C3%) was analyzed using the CodonW software. Additionally, G+C at the first (GC1s), second (GC2s) and third codon positions (GC3s) of each sequences were calculated by CodonW.

### Relative synonymous codon usage (RSCU)

Since the relative synonymous codon usage (RSCU) value directly reflects codon usage bias, it has been widely used to standardize the codon usage bias between genes or sets of genes that differ in their size and amino acid composition [19], which was proposed by *Sharpand Li* in 1986 [20]. It is considered that RSCU ignores the size and composition of amino acids [21]. RSCU was defined as the observed frequency of a particular amino acid to its standard frequency on all codons [22]. It is calculated using the following equation:

$$RSCU=\frac{g_{ij}}{\sum_{j}^{ni}g_{ij}}ni$$

where *g*_*ij*_ the observed number of codons for the amino acid and *ni* represents the degenerate numbers of a specific synonymous codon that ranges from 1 to 61 [23]. Normally, a higher RSCU value indicates a stronger codon usage bias. It is considered that no bias when the RSCU value is 1.0; if RSCU is more than or less than 1, there is considered to be a positive or negative codon usage bias [24], respectively. Additionally, codons with RSCU values >1.6 are over-represented and codons with RSCU values <0.6 are under-represented [25]. In this study, it was analyzed using the online software EMBOSS: cusp. (http://emboss.toulouse.inra.fr/cgi-bin/emboss/cusp). Additionally, the RSCU values of swine, which is the host of the CSFV, were downloaded from the codon usage database (http://www.kazusa.or.jp/codon/).

### Effective number of codons (ENC)

To quantify the magnitude of codon usage bias of each gene, the ENC value of each sequence was calculated, which is the best estimator of absolute synonymous codon usage bias [26]. The ENC was calculated using the following formula:

$$ENC=2+\frac{9}{{\bar{F}}_{2}}+\frac{1}{{\bar{F}}_{3}}+\frac{5}{{\bar{F}}_{4}}+\frac{3}{{\bar{F}}_{6}}$$

where F_(i = 2,3,4,6)_ represents the mean values of F_*i*_ with the i-fold degenerate amino acids. The F_*i*_ were calculated as the formula below:

$$F_{i}=\frac{n\sum_{j=1}^{i}{\left(\frac{n_{j}}{n}\right)}^{2}-1}{n-1}$$

where n stands for the total amount of the observed value of codons for a particular amino acid and *n*_*j*_ represents the observed numbers of the codon for that amino acid [27]. In contrast to RSCU, a lower ENC value denotes a higher codon usage bias. Previously, it was described as an ENC with a range of 20 to 61 [28].If just one synonymous codon encodes the corresponding amino acid, the ENC value is 20, while there is no codon usage bias with the ENC value of 61 [28]. Furthermore, it has been highlighted that an ENC value equal to or less than 35 is considered to be an extremely strong codon usage bias [26, 28].

To further determine the major factors affecting codon usage bias, an ENC-plot was generated which was completed with Graph Pad Prism6.0, with the ENC values plotted against the GC3s values. When the codon usage is only constrained by the GC3s, the observed ENC values will just lie on or around the standard curve. Otherwise, the observed values lying far lower than the standard curve, demonstrated that excepting for mutation pressure, other factors, such as natural selection, contribute to the codon usage pattern [27]. The standard ENC values were calculated using the equation:

$${ENC}_{expected}=2+s+\frac{29}{\left(s^{2}+{\left(1-s\right)}^{2}\right)}$$

where s stands for the occurrence of G+C of synonymous codons in the third codon position.

### General average hydropathicity (Gravy) and aromaticity (Aroma) indices

In analyzing the natural selection for shaping the codon usage bias of the CSFV E2 gene, two indices, including Gravy and Aroma scores, were involved in this study. They were analyzed by CodonW and signified the frequencies of hydrophobic and aromatic amino acids, respectively. Thus, the variation of the two indices reflects the amino acid usage [29]. A higher Gravy or Aroma value suggests a more hydrophobic or aromatic amino acid product.

### Principal component analysis (PCA)

PCA, a multivariate statistical approach in codon usage analysis, which plots axis1 against axis2 with the first two axes accounting for most of the component, was widely used to analyze the major trend in codon usage patterns among the different CSFV strains [30]. In the PCA, the RSCU values of each CSFV strain were distributed into a 59-dimensional vector corresponding to the 59 synonymous codons, excluding the codons of AUG, UGG and terminal codons. Thus, RSCU values were transformed into uncorrelated variables [31]. The PCA combined with the correlation analysis effectively demonstrated the factors influencing codon usage bias.

### Neutral evolution analysis

In investigating the varying roles of mutational pressure and natural selection in shaping the codon usage bias of the E2 gene of the CSFV, a neutrality plot was performed with GC12 as ordinate and GC3 as abscissa [32]. In the neutrality plot, each dot represents an independent CSFV strain. In general, if the slope of the regression line was 1, it was considered that the complete effect of neutrality constrains, while a slope of 0 was indicative of complete selective constrains [33].

### Statistical analysis

Using the statistical software Graph Pad Prism 6.0 with one-way analysis of variance (ANOVA) methods, a correlation analysis was carried out. The figures correlated to this study were drawn by Graph Pad Prism 6.0 and Origin 8.0.

## Results

### Recombination analysis

Previous study reported that possible recombination events influence the codon usage bias of genomes or genes [34, 35]. To detect whether the potential recombination exists in this study, a recombination analysis was performed. The results showed that all of the seven methods demonstrated that there were no recombinant events among the 140 coding sequences of CSFV E2 gene.

### Nucleotide composition of the CSFV E2 gene

In the present study, 140 coding sequences of E2 were analyzed to discover the codon usage of CSFV, including 98 sequences from China, 10 sequences from India, 6 from Bulgaria, 5 from Lithuania, 3 from Germany, Brazil and Romania, 2 from Viet Nam, and Croatia, and 1 from Italy, Hungary, Serbia, Latvia, Nepal, Slovakia, Switzerland and South Africa. The total number of employed codons were 156660 in this study. The composition properties of the CSFV strains are shown in S2 Table. The results revealed that the mean values of C%, G%, U%, and A% were 21.82%, 26.99%, 22.78%, and 27.72%, with a SD of 0.43, 0.42, 0.46 and 0.36, respectively, suggesting that all the compositions of the four kinds of nucleotides were less than 30%, and the A% was the most abundant, however, with subtle differences.

To further insight into the potential role of nucleotide content in shaping the codon usage pattern of CSFV E2 gene, the codon composition in the third position (A3, U3, G3, C3, and GC3) were calculated. It's revealed that among the codon compositions, the C3% was the highest with the mean value of 34.82%. As well as the GC3% fluctuated from 51.2% to 56.6%, with mean value of 53.89%. Demonstrating that C-ended codon might be preferred over A/U and G ended codons in the CSFV sequences, further confirming that although the difference among the total content of the four nucleotides were slight, there exists usage bias in the third position on the codons, thus, the nucleotide composition influenced the CSFV E2 gene.

### Relative synonymous codon usage (RSCU) and effective number of codon (ENC) in the CSFV E2 gene

RSCU values of 59 synonymous codons, excluding AUG and UGG, which just encode one amino acid, were calculated to explore the variation of synonymous codon usage in the CSFV E2 gene, which are listed in Table 1. The results showed that, among the 18 abundantly used synonymous codons, seven preferred codons terminated with C, which were TGC for Cys, GAC for Asp, CAC for His, TAC for Tyr, ACC for Thr, AGC for Ser, and AAC for Asn, and five optional codons terminated with A and G, respectively. Only one codon ended with U, which was UUU encode for Phe. It is interesting that codons ending with C were the most frequently employed among the eighteen synonymous codons, which was in according with the result of C being the most abundant among the third position of the four kinds of nucleotides, indicating that nucleotide bias was displayed in the CSFV E2 gene. Thus, the preferred codons were influenced by compositional constraints.

**Table 1:** Overall RSCU of the 140 collected sequences of the E2 gene of the CSFV.

Amino acid	Codon	RSCU/CSFV	RSCU/Swine	Amino acid	Codon	RSCU/CSFV	RSCU/Swine
Ala	GCA	**1.5624**	0.7386	Asn	AAC	**1.0105**	**1.2133**
	GCC	0.7196	**1.801**		AAT	0.9895	0.7867
	GCG	0.6969	0.5057	Pro	CCA	**1.1913**	0.9423
	GCT	1.0211	0.9546		CCC	1.1671	**1.4563**
Cys	TGC	**1.4769**	**1.2152**		CCG	0.5198	0.5536
	TGT	0.5231	0.7848		CCT	1.1219	1.0478
Asp	GAC	**1.1755**	**1.1975**	Gln	CAA	**1.3493**	0.441
	GAT	0.8245	0.8025		CAG	0.6507	**1.559**
Glu	GAA	0.9219	0.7256	Arg	AGA	2.4336	1.1155
	GAG	**1.0781**	**1.2744**		AGG	**3.1584**	1.2347
Phe	TTC	0.891	**1.2132**		CGA	0.0144	0.6065
	TTT	**1.109**	0.7868		CGC	0.0456	**1.3105**
Gly	GGA	0.7459	0.9117		CGG	0.3456	1.2888
	GGC	0.819	**1.4644**		CGT	0.0024	0.444
	GGG	**1.4884**	1.0541	Ser	AGC	**1.6293**	**1.6238**
	GGT	0.9468	0.5698		AGT	1.1627	0.7713
His	CAC	**1.5302**	**1.2946**		TCA	1.4124	0.7226
	CAT	0.4698	0.7054		TCC	1.2409	1.5021
Ile	ATA	**1.7028**	0.4208		TCG	0.0984	0.3897
	ATC	0.6896	**1.6697**		TCT	0.4565	0.9905
	ATT	0.6076	0.9095	Thr	ACA	1.1745	0.9145
Lys	AAA	0.7549	0.7603		ACC	**1.4914**	**1.6803**
	AAG	**1.2451**	**1.2397**		ACG	0.3478	0.5725
Leu	CTA	**1.5389**	0.3311		ACT	0.9864	0.8327
	CTC	0.7387	1.3475	Val	GTA	0.9328	0.3385
	CTG	1.4055	**2.6776**		GTC	0.9765	1.0646
	CTT	0.4511	0.6505		GTG	**1.5439**	**2.0308**
	TTA	0.6158	0.3195		GTT	0.5468	0.5661
	TTG	1.2499	0.6738	Tyr	TAC	**1.3893**	**1.2685**
					TAT	0.6107	0.7315

The preferentially used codons and RSCU values for E2 gene of the CSFV and swine are in bold.

Additionally, the ENC values were also employed to estimate the degree of codon usage bias of the CSFV strains [26]. The ENC values ranged from 49.74 to 55.7, with an average ±SD of 52.33±1.25, which revealed a relatively instable change. Furthermore, a higher ENC value of all the 140 CSFV strains indicated a significantly lower (ENC>40) codon usage bias.

### The role of mutational pressure in shaping codon usage bias

#### ENC-plot

**Fig 1:**
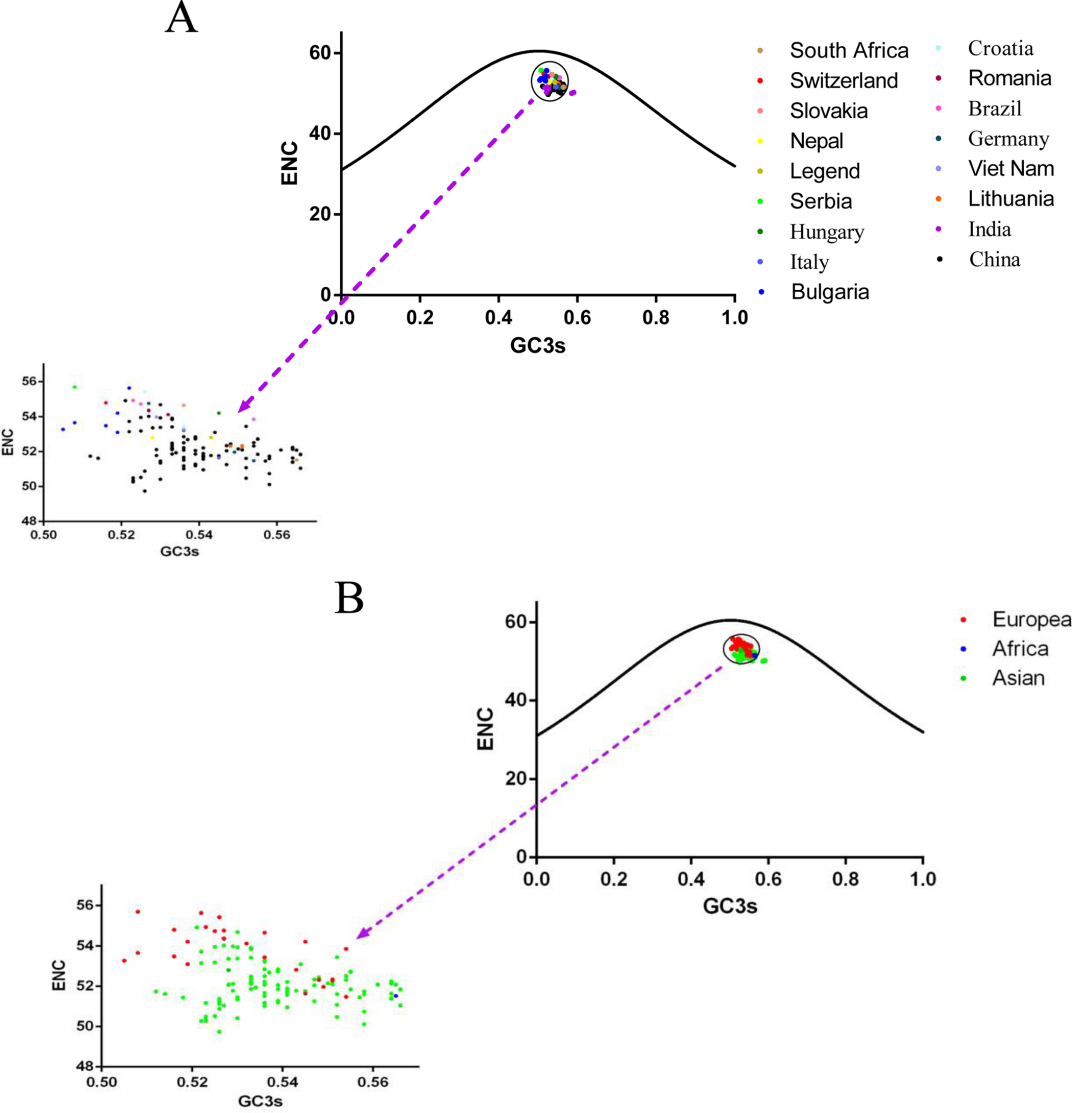
ENC plots displaying the relationships between ENC and the GC content at the third codon position (GC3s) in relation to geographical distribution. (A) isolated countries, (B) isolated continents, depicted in different colors. It's essential to note that the curve represent the expected relationship between ENC and GC3s, thus, justly GC3s contributes to the codon usage bias.

To further investigate the pattern of the synonymous codon usage, the ENC-plot, with ENC values plotted against GC3s values, were employed in this study (Fig 1). We observed that all points representing different strains were lower than the standard curve (Fig 1A). Additionally, even the strains isolated from the same country were not clustered together, particularly strains isolated from China. This implied that mutational pressure combined with other factors contributed to the codon usage bias in CSFV [32]. Further, as shown in Fig 1B, the ENC value of the European strains were higher than the strains belonging to Asian and Africa.

The correlation analysis between the nucleotide contents (A%, T%, G%, C%, and GC%) and the codon compositions (A3s, T3s, C3s, G3s, and GC3s) showed that all of them had a significant correlation with each other except for the relationship C3s with G. Moreover, the ENC value was remarkably correlated with the nucleotide contents with the P values far less than 0.01, indicating that the mutational pressure had an impact on the codon usage pattern of the E2 gene of the CSFV.

#### Principal component analysis (PCA)

**Fig 2:**
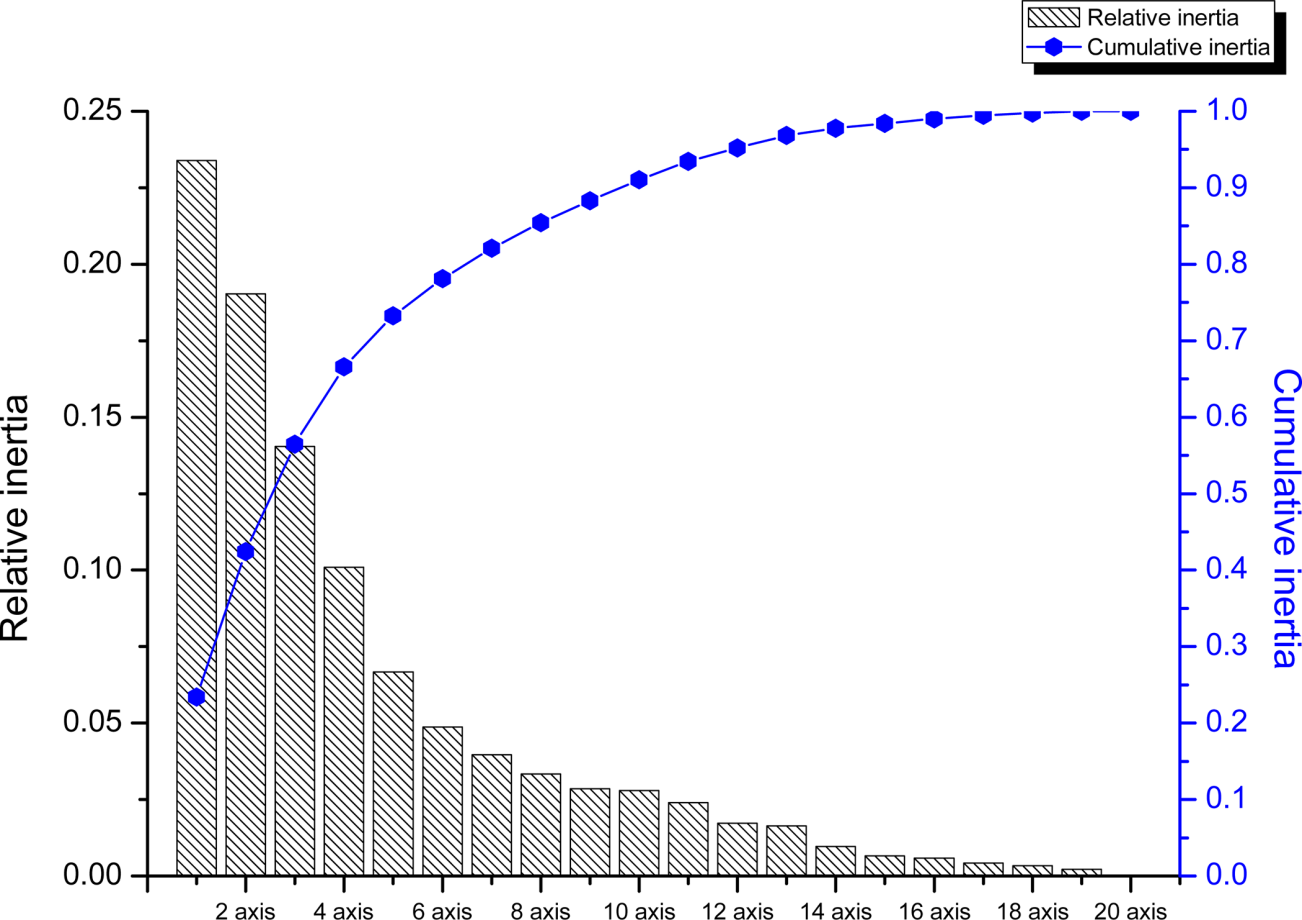
The tendency of codon usage bias based on the first 20 axes. The figure was based on the relative and cumulative inertia of the first 20 factors, respectively. The relative inertia is represented by the bar chart and the cumulative inertia is represented by the curve chart.

**Fig 3:**
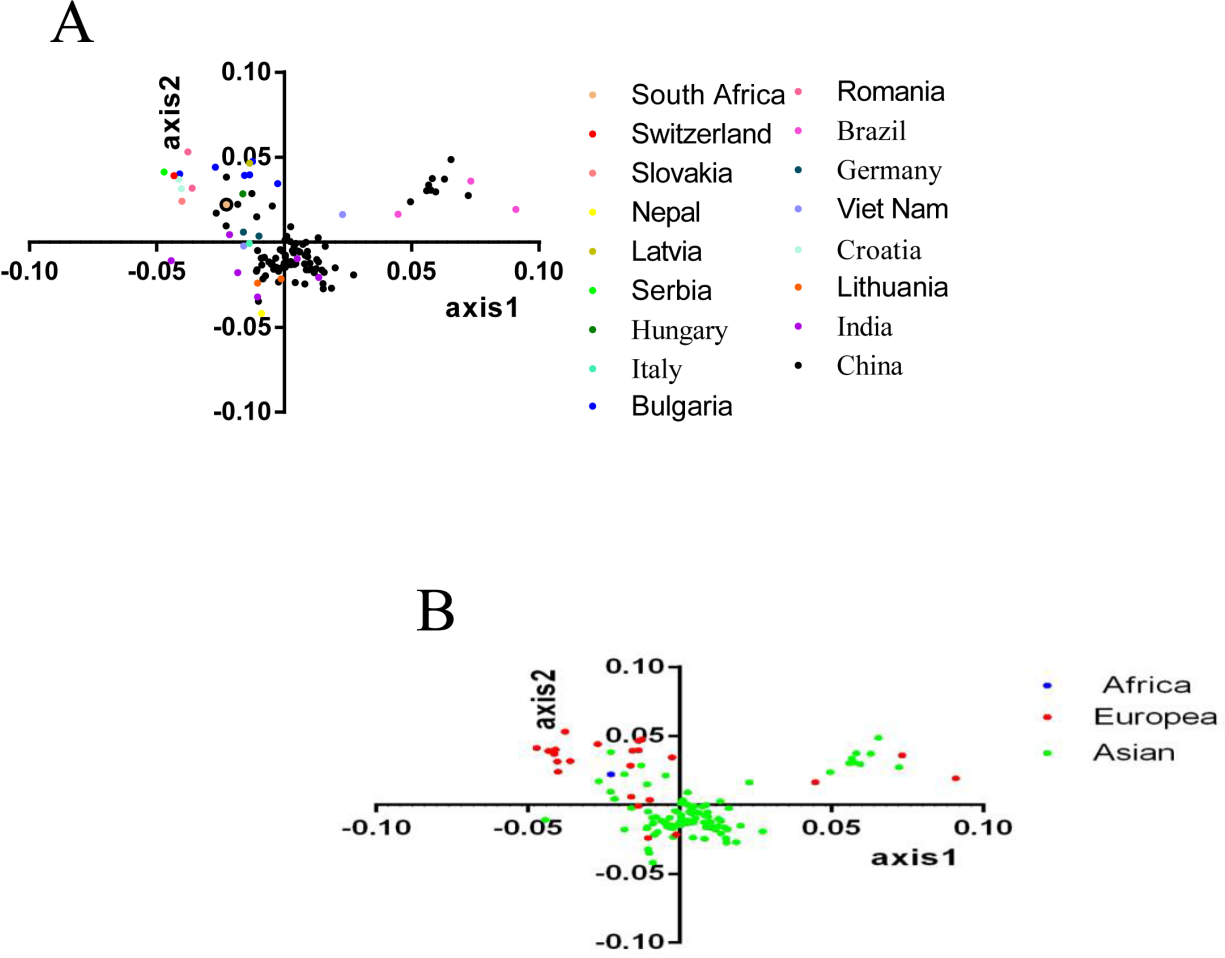
**PCA analysis, 1st axis plotted against 2nd axis according to the geographical distribution** (A) isolated countries; (B) isolated continents. Different geographical distributions are represented by different colors.

To further decrypt the trends of codon usage pattern in CSFV strains, a PCA was performed, which emphasized the corresponding distribution of the 59 variations [19]. The distributions of each vector are displayed in Fig 2, which were constructed by the software Origin8.0. The PCA results showed that among all the variations, the percentage of the first principal axis was 23.39% and the second axis, third axis and forth axis account for 19.03%, 14.05%, and 10.10% of all the variations (Fig 2), respectively. This revealed that the first four axes accounted for 66.58%. Additionally, the first and second axes play a major role among all the 59 variations, which was namely a tendency for codon usage bias. Therefore, a plot of the 1st axis and the 2nd axis of the isolated strains according to the geographical distribution was drawn (Fig 3). This explained that the different isolated countries with CSFV are dispersed, and far away from the origin, confirming mutation pressure. Other factors also played a role in shaping the codon usage bias of the E2 gene of the CSFV (Fig 3A). It is essential to denote that the majority of strains isolated from China were located near the origin, with only several China strains showing diversity, indicating that, compared with the other sixteen isolated countries, China strains contributed to mutation pressure to a large degree. Additionally, the majority of Asian strains were clustered together near the origin, suggesting that, compared with other strains, mutational bias was a major factor in shaping codon usage of the E2 gene of the CSFV Asian strains.

The correlation analysis between the codon compositions and the 1st axis and 2nd axis signified the codon compositions that were significantly correlated with the 1st axis and 2nd axis Table 2. These results thus confirmed that mutational pressure contributed to the codon usage bias of the CSFV E2 gene.

**Table 2:** The correlation analysis of nucleotide composition, Axis1, Axis2, Gravy, Aroma, nucleotide at the third position and ENC.

	A	C	G	U	ENC	Gravy	Aromo
GC3s	-0.6569**	0.8603**	0.5456**	-0.8025**	-0.3746**	0.1292	9.823E-05
T3s	0.3177**	-0.8897**	-0.2737**	0.8451**	0.4411**	-0.1129	-0.1399
C3s	-0.1677**	0.8777**	0.0064	-0.7076**	-0.2517**	0.0182	0.0556
A3s	0.8242**	-0.2887**	-0.6836**	0.2571**	0.0687	-0.1172	0.2176**
G3s	-0.7564**	0.1852**	0.8096**	-0.3257**	-0.2661**	0.1592*	-0.0877
GC	-0.7755**	0.7893**	0.7861**	-0.8609**	-0.4533**	0.1009	0.1124
Axis1	-0.2450**	-0.1166	0.3444**	-0.0075	-0.2931**	0.2462**	0.0625
Axis2	0.5032**	-0.3962**	-0.6318**	0.5565**	0.4294**	-0.0703	-0.1046

*P<0.05

**P<0.01

### The role of natural selection in the codon usage bias of CSFV E2 gene

It's considered that the relationship between Gravy, Aroma and axis1, axis2, GC3s, GC could explain the role of natural selection in shaping codon usage bias [35], therefore, the correlation analysis was employed to estimate the relationship between codon usage bias and the score of Gravy and Aroma Table 2, which demonstrated the influence of natural selection. The results revealed that Gravy significantly correlated with axis1, with P<0.01, while others were not correlated with Gravy and Aroma, revealing that natural selection shaped the codon usage pattern of the CSFV E2 gene with a sight role.

However, the RSCU of the CSFV and the viral host were compared in this study, which is listed in the Table 1. It is interesting that, among the 18 frequently used synonymous codons, 10 were commonly used in both CSFV and the viral host, highlighting that natural selection forced the CSFV to adapt to the natural host. Thus, natural selection was obvious in shaping the synonymous codon bias.

### Neutrality plot analysis

**Fig 4:**
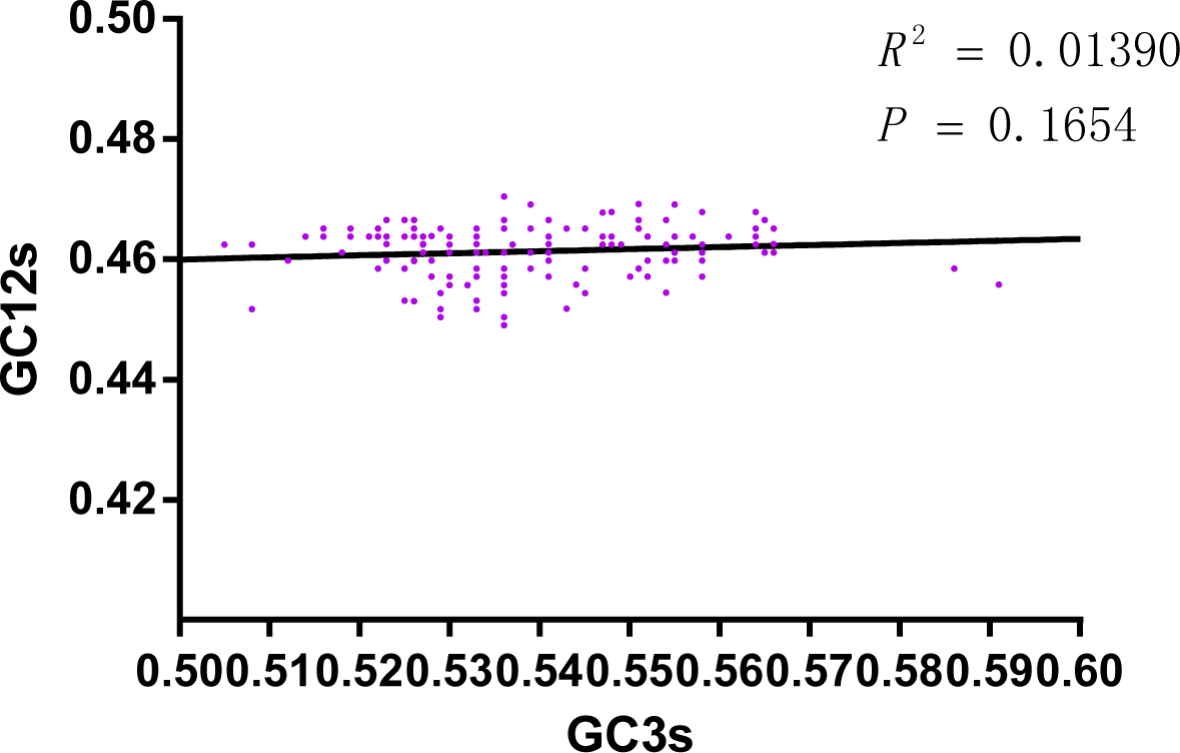
Neutrality analysis, with GC3s plotted against GC12s, displaying the key role between mutation pressure and natural selection.

It has been revealed that both mutation pressure and natural selection contributed to the codon usage bias of the CSFV. Therefore, the neutrality plot represents the relationship between the GC12s and the GC3s (Fig 4), which was employed to discern the directional mutation pressure *vs* natural selection that shapes codon usage in the E2 gene of the CSFV [27]. The analysis showed that the GC3s was significantly correlated with GC12s (r = 0.357, P<0.0001). However, the correlation coefficient of the neutrality plot was 0.1043, highlighting the relative neutrality was 10.43%. In other words, the relative selective constraint was 89.57% as calculated previously [32, 34]. It is interesting that, compared with mutation pressure, natural selection is dominant in shaping the codon usage pattern of the CSFV E2 gene.

## Discussion

Different from most of the DNA viruses, the zoonotic RNA viruses, such as influenza viruses and coronaviruses, have highly susceptible to recombination and cross species transmission [36–39]. CSFV, a single stranded, positive and non-segmented virus [40], encodes non-structural proteins, including Npro, P7, NS2, NS3, NS4A, NS4B, NS5A, NS5B, and structural proteins, including C, Erns, E1, E2 [41, 42]. Previous studies indicated that, in the analysis of genomic sequencing, variations were frequently found in CSFV, 5'NTR, E2 and NS5B [43–45]. Additionally, the E2 glycoprotein is remarkable in affecting viral virulence and escaping immune responses [46]. However, the genetic bias of E2 glycoprotein in CSFV has not been studied.

It has been previously shown that codon usage bias of vaccinated and non-vaccinated CSFV strains were different and the vaccination may influence the evolution of CSFV [47]. It is considered that codon usage bias of different species and different genes, even belonging to a virus, is different during evolution [48–50]. Additionally, the degree of codon usage is comprised of the following factors: mutation pressure, translational selection, the abundance of tRNAs, secondary mRNA structure, and gene length [10]. Two factors mainly affect codon usage; that is, mutation pressure and selection pressure [51, 52]. Further, in codon usage analysis on some DNA viruses and RNA viruses, mutational bias is a decisive factor compared with natural selection, such as the FMDV [53] and Tembusu virus [54]; however, in the codon usage analysis on PEDV, it was revealed that natural selection dominates over mutation pressure [10].

In this study, we firstly showed that codon usage bias of the E2 gene of the CSFV was higher, with ENC values ranging from 49.74 to 55.7, mean ±SD of 52.33±1.25, compared with the other members of pestivirus BVDV with an ENC of 51.43. It has been reported that ENC values of the complete genome of the CSFV ranged from 51.07 to 52.15, with a mean±SD of 51.7±0.26 [55], while the ENC values of E2 was wider, which might contribute to the characteristics of immunoprotective antigens. Additionally, other RNA viruses, such as FMDV [11], have a mean value of 51.42, and the SARS [56] virus, has a mean value of 48.99.

In the analysis investigating the codon usage of synonymous codons, it was shown that RSCU, based on 59 sense codons and among the 18 frequently exerted synonymous codons, C terminated codons were most abundant. Next, were A, G and U, while the nucleotide content analysis decrypted that among the four nucleotides, A%was the highest, which suggested that the nucleotide composition constrains synonymous codon usage of the E2 gene of the CSFV. Additionally, ENC-plot analysis (Fig 1) and PCA analysis (Fig 3), based on the geographical distribution (isolated country and continent), were performed. Fig 1 shows that the value of each 140 independent strains was lower than the standard value, and strains isolated from three continents were clustered mainly in three groups, which was, according to the survey, that the prevalence of CSFV was mainly in Asian [57] and European [58] countries. The PCA analysis (Fig 3) demonstrated that the majority of Asian strains clustered near the origin, and European strains were disparate, which might be due to Asian countries being old epidemic areas, while European countries are actively prevalence areas. Then, the correlation between Aroma, Gravy and codon usage indices and among the 18 frequently used synonymous codons, 10 were commonly used in both CSFV and the viral host indicated the role of natural selection. The two main factors contributed to the codon usage of the E2 gene of the CSFV to different degrees. Thus, the neutrality analysis, GC3s plotted against GC12s, decrypted that GC3s had no correlation with GC12s. Furthermore, the extent of mutational bias occupied only 10.43%, and otherwise the translational selection was 89.57%, highlighting that in natural selection *vs* mutation pressure in shaping codon usage of the E2 gene of the CSFV, natural selection had a significant role.

In conclusion, 140 sequences of the E2 gene were exerted in this study to explore the codon usage of CSFV, which substantiated the evolutionary process of the CSFV; however, the relatively small selected sequences may be not fully representative, which might provide only slight evidence of CSFV. Additionally, there exists limitation that only little information is provided regarding other gene or coding sequence of CSFV. Taking into account the CSFV growing epidemic situation, and the threat to the pig farming industry, in the future, more epidemiological survey to examining the factors that resulted in the outbreak and evolution of this virus is needed.

## Supporting information

S1 Table The detail information of the 140 collected sequences of E2 gene of CSFV. (DOCX)

S2 Table The nucleotides composition of the 140 sequences of E2 gene of CSFV. (DOCX)
