# Supplementary material for: Comprehensive analysis of the codon usage patterns in the envelope glycoprotein E2 gene of the classical swine fever virus
Source: PLoS One. 2017 Sep 7;12(9):e0183646. doi: 10.1371/journal.pone.0183646 (PMC5589121; doi:10.1371/journal.pone.0183646)
Supplement: S1 Table — (DOCX) [file pone.0183646.s001.docx]

**S1 Table. The detail information of the 140 collected sequences of E2 gene of CSFV.**

| **Number** | **Strain name** | **Country** | **Year** |
| --- | --- | --- | --- |
| KX257416 | CH/JX/NC/01 | China | 2001 |
| EF683623 | SX-04 | China | 2004 |
| EF683605 | HuZ1-04 | China | 2004 |
| FJ456870 | JX-04 | China | 2004 |
| EF683607 | HZ1-04 | China | 2004 |
| EF683608 | HZ2-04 | China | 2004 |
| FJ456875 | HZ2-04 | China | 2004 |
| EF683606 | HuZ2-05 | China | 2005 |
| FJ456869 | HuZ1-05 | China | 2005 |
| EF683621 | SH2-05 | China | 2005 |
| EF683616 | JX-05 | China | 2005 |
| EF683617 | LS-05 | China | 2005 |
| FJ456871 | HZ-05 | China | 2005 |
| EF683613 | JX1-06 | China | 2006 |
| EF683615 | JX4-06 | China | 2006 |
| KX759643 | HuN06 | China | 2006 |
| EF683618 | QZ1-06 | China | 2006 |
| EF683619 | QZ2-06 | China | 2006 |
| FJ456867 | HZ1-06 | China | 2006 |
| FJ456872 | SX2-06 | China | 2006 |
| FJ456873 | JX2-06 | China | 2006 |
| FJ456865 | SH2-07 | China | 2007 |
| FJ456866 | HZ1-07 | China | 2007 |
| FJ456868 | JX-07 | China | 2007 |
| FJ456876 | QZ-07 | China | 2007 |
| EF683610 | HZ2-07 | China | 2007 |
| EF683612 | JS-07 | China | 2007 |
| EF683622 | SX3-06 | China | 2008 |
| KJ661548 | Guizhou | China | 2008 |
| HQ380236 | CSFV-PR-2008 | China | 2008 |
| FJ582642 | HZ1-08 | China | 2008 |
| FJ582643 | HZ2-08 E2 | China | 2008 |
| FJ582644 | FY-08 | China | 2008 |
| FJ598609 | QZ1-08 | China | 2008 |
| FJ598611 | C-strain-ZJ | China | 2008 |
| FJ598612 | Shimen-ZJ | China | 2008 |
| FJ607779 | ZS1-08 | China | 2008 |
| FJ607780 | ZS2-08 | China | 2008 |
| HQ380232 | CSFV-DB-2009 | China | 2009 |
| HQ380245 | CSFV-ZQ-2010 | China | 2009 |
| HQ380238 | CSFV-FS-2009 | China | 2009 |
| HQ380240 | CSFV-NH-2009 | China | 2009 |
| KX759642 | SX(09) E2 gene | China | 2009 |
| HQ317681 | GDST1.2010 | China | 2010 |
| HQ380243 | CSFV-JY-2010 | China | 2010 |
| JN882005 | HNLY-11 | China | 2011 |
| JN886990 | HNZH-2011 | China | 2011 |
| JQ001833 | SDQS11 | China | 2011 |
| JQ001834 | SDQS11 | China | 2011 |
| JX898523 | HNCS-2011 | China | 2011 |
| KC809979 | 2-55/HeB-2011 | China | 2011 |
| KC809981 | 1-5/HeN-2011 | China | 2011 |
| KC809982 | 2-31/HeN-2011 | China | 2011 |
| KC809983 | 1-19/HeB-2011 | China | 2011 |
| KC809984 | 2-41/HeB-2011 | China | 2011 |
| KC809985 | 1-12/SD-2011 | China | 2011 |
| KC867687 | 2-9/SD-2011 | China | 2011 |
| KT853103 | GD52/2011 | China | 2011 |
| KT853104 | GD53/2011 | China | 2011 |
| KT853106 | GD156/2011 | China | 2011 |
| KT853108 | GD191/2011 | China | 2011 |
| KT853109 | GD317/2011 | China | 2011 |
| KT853110 | GD318/2011 | China | 2011 |
| KT853111 | GD45/2011 | China | 2011 |
| KT853113 | GD12/2011 | China | 2011 |
| KT853115 | GD143/2011 | China | 2011 |
| KC809986 | 1-11/HeN-2012 | China | 2012 |
| KU375252 | JLHD2012 | China | 2012 |
| JX898525 | HNYY-2012 | China | 2012 |
| KC597187 | CSFV/2.1/dp/HeNan65/2012/Henan | China | 2012 |
| KC809980 | 1-21/HeB-2012 E2 | China | 2012 |
| KC867688 | 2-59/HeN-2012 | China | 2012 |
| KC867689 | 3-37/HeB2012 | China | 2012 |
| KT953592 | SDTA1-13 | China | 2013 |
| KU375253 | BJSN2013 | China | 2013 |
| KT953604 | SDLY-14 | China | 2014 |
| KU375249 | HLJWC2014-1 | China | 2014 |
| KU375250 | HLJWC2014-2 | China | 2014 |
| KU375251 | JLHN2014 | China | 2014 |
| KU375257 | HLJAC2014 | China | 2014 |
| KU375259 | JLFY2014-2 | China | 2014 |
| KU375260 | HLJZZ2014 | China | 2014 |
| KU375262 | HLJB2014 | China | 2014 |
| KU375255 | HLJQH2015 | China | 2015 |
| KT953587 | SDJNi1-15 | China | 2015 |
| KT953590 | SDZB2-15 | China | 2015 |
| KU375263 | HLJBY2015 | China | 2015 |
| KT953594 | SDLY-15 | China | 2015 |
| KT953596 | SDJNi3-15 | China | 2015 |
| KT953597 | SDLW2-15 | China | 2015 |
| KT953599 | SDMZ1-15 | China | 2015 |
| KT953601 | SDJNi6-15 | China | 2015 |
| KT953603 | SD19-15 | China | 2015 |
| KU375254 | JLCL2015 | China | 2015 |
| KT953605 | SDZB-15 | China | 2015 |
| KT953607 | SDHZ-15 | China | 2015 |
| KT953609 | SDXLS-15 | China | 2015 |
| KT953611 | SDXT-15 | China | 2015 |
| KR054034 | IND/KAR/KDG | India | No Date |
| KR054036 | IND/KAR/BNG(URB2) | India | No Date |
| KR054038 | IND/KAR/BNG(URB3) | India | No Date |
| KR054040 | IND/KAR/MND | India | No Date |
| KR054042 | IND/KAR/BNG(URB4) | India | No Date |
| KR054045 | IND/KAR/KPL | India | No Date |
| KR054048 | IND/KAR/BDR | India | No Date |
| KR054051 | IND/KAR/BNG (RUR5) | India | No Date |
| LC000001 | ML/RRTC-B14 | India | 2013 |
| LC000002 | AS/Guwahati-G4 | India | 2012 |
| JQ411592 | CSF1055 | Lithuania | 2011 |
| JQ411594 | 862-3 | Lithuania | 2011 |
| JQ411597 | 862-16 | Lithuania | 2011 |
| JQ411599 | 919-7 | Lithuania | 2011 |
| JQ411601 | 940-10 | Lithuania | 2011 |
| KF233944 | CSF0609 | Bulgaria | 1997 |
| KX687718 | CSF0804 | Bulgaria | 2002 |
| KF233949 | CSF0853 | Bulgaria | 2006 |
| KF233951 | CSF086 | Bulgaria | 2007 |
| KF233957 | CSF1044 | Bulgaria | 2008 |
| KF233959 | CSF1051 | Bulgaria | 2009 |
| KP702208 | ND9 E2 gene | Viet Nam | 2014 |
| KP702210 | ND21 E2 gene | Viet Nam | 2014 |
| KX687720 | CSF0183 | Germany | 1993 |
| KX687712 | CSF0934 | Germany | 1989 |
| AY027673 | Paderborn | Germany | No Date |
| KX431229 | Caucaia/2003 | Brazil | 2003 |
| KX431231 | Caucaia/2004 | Brazil | 2004 |
| KX431233 | Macapa | Brazil | 2009 |
| KX687715 | CSF0631 | Romania | 1994 |
| KF233955 | CSF0892 | Romania | 2006 |
| KF297337 | CSF0890 | Romania | 2007 |
| KX687713 | CSF0818 | Croatia | 2002 |
| KF233953 | CSF0870 | Croatia | 2007 |
| AY027672 | Italy | Italy | No Date |
| KX687721 | CSF0088 | Hungary | 1992 |
| KF233960 | CSF1053 | Serbia | 2010 |
| KF233961 | CSF1070 | Latvia | 2013 |
| JX162241 | CSF1060 | Nepal | 2011 |
| KX687719 | CSF1040 | Slovakia | 2007 |
| KF233946 | CSF0736 | Switzerland | 2000 |
| HM190299 | Elsenburg from South Africa | South Africa | 2005 |
