# Supplementary material for: Comprehensive analysis of the codon usage patterns in the envelope glycoprotein E2 gene of the classical swine fever virus
Source: PLoS One. 2017 Sep 7;12(9):e0183646. doi: 10.1371/journal.pone.0183646 (PMC5589121; doi:10.1371/journal.pone.0183646)
Supplement: S2 Table — (DOCX) [file pone.0183646.s002.docx]

**S2 Table. The nucleotides composition of the 140 sequences of E2 gene of CSFV.**

| **Number** | **A%** | **C%** | **G%** | **U%** |
| --- | --- | --- | --- | --- |
| AY027672 | 27.88 | 21.89 | 27.17 | 23.06 |
| AY027673 | 27.79 | 22.16 | 27.17 | 22.88 |
| EF683605 | 27.61 | 21.98 | 27.44 | 22.97 |
| EF683606 | 27.79 | 21.81 | 27.44 | 22.97 |
| EF683607 | 27.70 | 22.16 | 27.08 | 23.06 |
| EF683608 | 27.79 | 22.34 | 27.26 | 22.61 |
| EF683610 | 27.26 | 22.61 | 27.97 | 22.16 |
| EF683612 | 27.26 | 22.70 | 27.97 | 22.07 |
| EF683613 | 27.88 | 22.52 | 27.35 | 22.25 |
| EF683615 | 27.52 | 22.61 | 27.70 | 22.16 |
| EF683616 | 27.79 | 22.25 | 27.26 | 22.70 |
| EF683617 | 27.79 | 22.34 | 27.44 | 22.43 |
| EF683618 | 27.61 | 22.61 | 27.52 | 22.25 |
| EF683619 | 27.35 | 22.61 | 27.88 | 22.16 |
| EF683621 | 27.52 | 22.07 | 27.61 | 22.79 |
| EF683622 | 27.35 | 22.34 | 27.88 | 22.43 |
| EF683623 | 27.44 | 22.16 | 27.79 | 22.61 |
| FJ456865 | 27.44 | 22.52 | 27.79 | 22.25 |
| FJ456866 | 27.35 | 22.52 | 27.97 | 22.16 |
| FJ456867 | 27.35 | 22.43 | 27.88 | 22.34 |
| FJ456868 | 27.79 | 22.43 | 27.44 | 22.34 |
| FJ456869 | 27.79 | 22.79 | 27.44 | 21.98 |
| FJ456870 | 27.61 | 22.70 | 27.52 | 22.16 |
| FJ456871 | 27.61 | 22.52 | 27.70 | 22.16 |
| FJ456872 | 27.70 | 22.25 | 27.52 | 22.52 |
| FJ456873 | 27.61 | 22.07 | 27.44 | 22.88 |
| FJ456875 | 27.97 | 21.98 | 27.17 | 22.88 |
| FJ456876 | 27.97 | 22.16 | 26.99 | 22.88 |
| FJ582642 | 27.97 | 22.07 | 27.26 | 22.70 |
| FJ607779 | 28.06 | 22.43 | 27.17 | 22.34 |
| FJ607780 | 27.97 | 22.43 | 27.35 | 22.25 |
| HM190299 | 27.44 | 22.34 | 27.44 | 22.70 |
| HQ317681 | 28.33 | 21.98 | 26.72 | 22.97 |
| HQ380232 | 28.42 | 21.45 | 26.90 | 23.24 |
| HQ380236 | 28.33 | 21.63 | 26.99 | 23.06 |
| HQ380238 | 28.42 | 21.72 | 26.90 | 22.97 |
| HQ380245 | 28.42 | 21.54 | 26.90 | 23.15 |
| JX898523 | 28.24 | 21.63 | 26.72 | 23.41 |
| JX898525 | 27.97 | 21.81 | 26.90 | 23.32 |
| KC597187 | 27.97 | 21.63 | 26.90 | 23.50 |
| KC809979 | 27.97 | 21.89 | 27.35 | 22.79 |
| KC809980 | 28.15 | 22.16 | 27.17 | 22.52 |
| KF233949 | 28.33 | 21.72 | 26.54 | 23.41 |
| KF233951 | 28.33 | 21.54 | 26.63 | 23.50 |
| KF233953 | 28.42 | 21.54 | 26.45 | 23.50 |
| KF233955 | 28.24 | 21.98 | 26.45 | 23.32 |
| KF233957 | 29.13 | 21.81 | 25.92 | 23.15 |
| KF233959 | 28.42 | 21.63 | 26.45 | 23.50 |
| KF233960 | 28.60 | 21.09 | 26.27 | 24.04 |
| KF233961 | 28.24 | 21.98 | 26.63 | 23.15 |
| KF297337 | 28.42 | 22.16 | 26.27 | 23.06 |
| KJ661548 | 28.33 | 21.54 | 27.08 | 23.06 |
| KT853106 | 27.17 | 21.81 | 27.61 | 23.41 |
| KT853108 | 28.15 | 21.89 | 26.72 | 23.24 |
| KT853109 | 27.26 | 21.89 | 27.70 | 23.15 |
| KT853110 | 27.26 | 21.89 | 27.70 | 23.15 |
| KT853111 | 27.70 | 21.63 | 27.52 | 23.15 |
| KT853113 | 27.61 | 22.43 | 27.26 | 22.70 |
| KT853115 | 27.61 | 22.43 | 27.26 | 22.70 |
| KT953587 | 27.79 | 21.45 | 27.52 | 23.24 |
| KT953590 | 28.24 | 22.07 | 27.17 | 22.52 |
| KT953592 | 27.88 | 21.81 | 27.35 | 22.97 |
| KT953594 | 27.88 | 21.81 | 27.35 | 22.97 |
| KT953596 | 27.97 | 22.25 | 27.17 | 22.61 |
| KT953597 | 27.97 | 21.89 | 27.26 | 22.88 |
| KT953599 | 27.97 | 21.98 | 27.35 | 22.70 |
| KT953601 | 27.97 | 21.81 | 27.08 | 23.15 |
| KT953603 | 27.44 | 21.81 | 27.35 | 23.41 |
| KT953604 | 27.97 | 21.89 | 27.35 | 22.79 |
| KT953605 | 28.06 | 22.16 | 27.26 | 22.52 |
| KT953607 | 27.79 | 22.07 | 27.44 | 22.70 |
| KT953609 | 28.06 | 21.89 | 27.26 | 22.79 |
| KU375250 | 27.88 | 21.63 | 27.35 | 23.15 |
| KU375251 | 27.79 | 21.89 | 27.44 | 22.88 |
| KU375252 | 27.88 | 21.89 | 27.44 | 22.79 |
| KU375253 | 28.06 | 21.98 | 27.35 | 22.61 |
| KU375254 | 27.79 | 21.72 | 27.44 | 23.06 |
| KU375255 | 27.79 | 21.81 | 27.44 | 22.97 |
| KU375257 | 27.88 | 21.81 | 27.35 | 22.97 |
| KU375259 | 28.15 | 22.07 | 27.26 | 22.52 |
| KU375260 | 27.97 | 21.72 | 27.35 | 22.97 |
| KU375262 | 28.42 | 21.72 | 26.72 | 23.15 |
| KU375263 | 27.88 | 21.81 | 27.35 | 22.97 |
| KX257416 | 27.52 | 21.81 | 27.61 | 23.06 |
| KX431229 | 27.70 | 21.36 | 27.26 | 23.68 |
| KX431231 | 27.52 | 21.45 | 27.26 | 23.77 |
| KX431233 | 27.26 | 22.07 | 27.61 | 23.06 |
| KX687712 | 27.88 | 22.07 | 26.99 | 23.06 |
| KX687713 | 28.78 | 21.98 | 26.18 | 23.06 |
| KX687715 | 28.69 | 21.98 | 26.18 | 23.15 |
| KX687718 | 28.33 | 21.36 | 26.54 | 23.77 |
| KX687719 | 28.06 | 21.81 | 26.72 | 23.41 |
| KX687720 | 28.69 | 21.81 | 26.27 | 23.24 |
| KX687721 | 28.42 | 22.25 | 26.45 | 22.88 |
| KX759642 | 27.52 | 22.07 | 27.26 | 23.15 |
| KX759643 | 27.70 | 22.34 | 27.26 | 22.70 |
| LC000001 | 27.44 | 23.68 | 27.17 | 21.72 |
| LC000002 | 27.52 | 23.77 | 27.08 | 21.63 |
| KT953611 | 27.88 | 22.07 | 27.44 | 22.61 |
| KU375249 | 27.88 | 21.63 | 27.35 | 23.15 |
| KT853104 | 28.15 | 21.89 | 26.72 | 23.24 |
| KT853103 | 28.15 | 21.81 | 26.72 | 23.32 |
| FJ582643 | 27.61 | 22.52 | 27.61 | 22.25 |
| FJ582644 | 27.61 | 22.61 | 27.61 | 22.16 |
| FJ598609 | 28.06 | 22.52 | 27.17 | 22.25 |
| FJ598611 | 27.26 | 21.09 | 27.70 | 23.95 |
| FJ598612 | 28.24 | 21.63 | 26.90 | 23.24 |
| JQ001833 | 27.79 | 21.63 | 26.99 | 23.59 |
| JQ001834 | 27.97 | 21.81 | 27.08 | 23.15 |
| JQ411592 | 27.97 | 22.25 | 26.99 | 22.79 |
| JQ411594 | 27.97 | 22.34 | 26.99 | 22.70 |
| JQ411597 | 27.97 | 22.34 | 26.99 | 22.70 |
| JQ411599 | 28.06 | 22.34 | 26.90 | 22.70 |
| JQ411601 | 27.97 | 22.25 | 26.99 | 22.79 |
| JX162241 | 27.52 | 21.36 | 27.79 | 23.32 |
| KP702208 | 27.88 | 21.98 | 26.90 | 23.24 |
| KP702210 | 27.61 | 20.91 | 27.61 | 23.86 |
| HQ380240 | 28.60 | 21.72 | 26.72 | 22.97 |
| HQ380243 | 28.06 | 21.00 | 26.81 | 24.13 |
| KR054034 | 28.06 | 21.27 | 27.35 | 23.32 |
| KR054036 | 28.06 | 21.54 | 27.35 | 23.06 |
| KR054038 | 28.06 | 21.45 | 27.35 | 23.15 |
| KR054040 | 28.15 | 21.98 | 26.63 | 23.24 |
| KC809984 | 27.97 | 21.89 | 27.17 | 22.97 |
| KR054048 | 27.97 | 21.45 | 27.44 | 23.15 |
| KR054051 | 27.97 | 21.45 | 27.44 | 23.15 |
| KC867688 | 27.97 | 21.89 | 27.17 | 22.97 |
| KC867689 | 28.15 | 21.98 | 26.81 | 23.06 |
| KF233944 | 28.33 | 21.63 | 26.36 | 23.68 |
| KF233946 | 28.69 | 21.72 | 26.18 | 23.41 |
| KR054045 | 28.06 | 21.54 | 27.35 | 23.06 |
| KC809985 | 27.70 | 21.54 | 27.44 | 23.32 |
| KR054042 | 27.79 | 21.36 | 27.70 | 23.15 |
| KC809986 | 27.52 | 21.98 | 27.17 | 23.32 |
| KC867687 | 27.52 | 21.81 | 27.52 | 23.15 |
| KC809982 | 27.88 | 22.16 | 27.17 | 22.79 |
| KC809983 | 27.88 | 21.81 | 27.26 | 23.06 |
| KC809981 | 27.88 | 22.16 | 27.26 | 22.70 |
| JN882005 | 28.15 | 21.72 | 26.81 | 23.32 |
| JN886990 | 28.33 | 21.72 | 26.63 | 23.32 |
| Mean | 27.72 | 21.82 | 26.99 | 22.78 |
| SD | 0.36 | 0.43 | 0.42 | 0.46 |
